# From body composition to reflux esophagitis: an interpretable machine learning model based on CT-derived features

**DOI:** 10.3389/fphys.2026.1788389

**Published:** 2026-05-08

**Authors:** Tianyi Wang, Lu Chen, Yajie Li, Qi Zhang, Xilong Ou, Qin Lu

**Affiliations:** 1 Medical Faculty, Southeast University, Nanjing, Jiangsu, China; 2 Department of Gastroenterology, The Affiliated Zhongda Hospital of Southeast University, Nanjing, Jiangsu, China; 3 Department of Gerontology, The Affiliated Zhongda Hospital of Southeast University, Nanjing, Jiangsu, China

**Keywords:** body composition, IMAT, low SMI, machine learning, random forest, reflux esophagitis

## Abstract

**Background:**

The prevalence of gastroesophageal reflux disease (GERD) has been increasing in China. Previous studies link sarcopenia and visceral adiposity to GERD, but most models lack CT-based body composition data. This study aims to improve the identification of Reflux esophagitis (RE) by applying machine learning (ML) to third lumbar vertebra cross-sectional CT (L3-CT) images for quantitative analysis of muscle and fat mass.

**Methods:**

Participants underwent comprehensive abdominal CT and gastroscopy. Body composition parameters, including skeletal muscle mass (SM), total fat mass (FM), fat-free mass (FFM), visceral adipose tissue (VAT), subcutaneous adipose tissue (SAT), and intermuscular adipose tissue (IMAT), were derived from the L3-CT images. Six ML models were developed: Random Forest (RF), Extreme Gradient Boosting (XGBoost), Logistic Regression (LR), K-Nearest Neighbors (KNN), Support Vector Machine (SVM), and Artificial Neural Network (ANN). Model performance was evaluated using the area under the receiver operating characteristic curve (AUROC). Shapley Additive exPlanations (SHAP) values were used to interpret the RF model.

**Results:**

A total of 324 subjects were included, with 135 diagnosed with RE. The prevalence of low skeletal muscle mass index (low SMI) was significantly higher in the RE group compared to controls (52.6% vs. 36.5%, *P* < 0.01). The top six variables in the RF importance matrix were IMAT, visceral-to-subcutaneous fat ratio (VSR), age, VAT, SAT, and hiatal hernia. In the validation set, RF (AUC = 0.829, 95% CI: 0.731–0.905) and LR (AUC = 0.829, 95% CI: 0.736–0.909) demonstrated the best discriminative performance for RE. SHAP summary plots illustrated the positive and negative contributions of the top 20 features, while SHAP dependence plots explained the impact of individual variables on the RF model output.

**Conclusion:**

This study reveals a significant association between low SMI and RE. ML models identified key body composition factors, providing insights for targeted screening and clinical assessment.

## 1 Introduction

The prevalence of gastroesophageal reflux disease (GERD) in China is 3.8% and is rising annually (Chen et al., 2005). GERD is characterized by the reflux of gastric contents into the esophagus or beyond, leading to typical esophageal symptoms such as heartburn, dysphagia, and chest pain, as well as extraesophageal manifestations such as cough, hoarseness, nausea, and asthma (Klenzak et al., 2018; Tack and Pandolfino, 2018). Reflux esophagitis (RE) is a major subtype of GERD, presenting as endoscopic evidence of esophageal mucosal inflammation and erosion, accompanied by varying degrees of gastrointestinal symptoms (Sun et al., 2019; Mittal et al., 2021). Persistent RE not only reduces patients’ quality of life but also increases the risk of complications such as Barrett’s esophagus and esophageal adenocarcinoma.

Various factors have been identified as contributing to the development of RE. A large European Mendelian randomization cohort study integrating genome-wide association analysis demonstrated significant causal associations between obesity, type 2 diabetes, and smoking with the onset of GERD; further analysis suggested that central obesity and caffeine intake may serve as independent risk factors for GERD (Yuan and Larsson, 2022). An earlier cross-sectional cohort study indicated an association between metabolic syndrome (MS), characterized by visceral obesity, and RE (OR = 1.42, 95% CI: 1.26–1.60). Further analysis of MS components revealed that VAT was an independent risk factor for RE (OR = 1.61, 95% CI: 1.10–2.36), whereas SAT showed no correlation (Chung et al., 2008). A recent study utilizing deep neural network-based CT analysis confirmed a significant positive association between VAT area and volume and RE risk (Han et al., 2024). Collectively, these findings collectively indicate that fat content and obesity-related metabolic syndrome are pivotal risk factors for GERD. Furthermore, a significant relationship has been demonstrated between decreased skeletal muscle mass, sarcopenia, sarcopenic obesity, and GERD in previous studies (Kim et al., 2019; Hu et al., 2024).

However, previous studies on the association between sarcopenia, sarcopenic obesity, and GERD have primarily defined sarcopenia based on skeletal muscle mass estimated using bioelectrical impedance analysis. Recent research has confirmed that CT and MRI provide more accurate assessments of muscle mass and composition, establishing them as the gold standard for body composition analysis (Muller et al., 2016, 2002). Moreover, all the before mentioned studies relied on logistic regression models, which require statistical assumptions about linear relationships between variables and outcomes. These models lack the capacity to automatically capture nonlinear patterns and depend on manual feature engineering to handle interactions. This constraint may lead to the exclusion of variables due to statistical assumptions, potentially reducing predictive accuracy. Conversely, machine learning (ML) techniques do not require predefined assumptions about input variables or their relationships with outcomes. By autonomously selecting features, modelling nonlinear relationships, and applying regularization strategies, ML significantly enhances generalizability (Jiang et al., 2017; Rajkomar et al., 2019). Despite the potential limitations of interpretability in ML models, this study addresses this challenge by using Shapley Additive exPlanations (SHAP) values to quantify the contribution of each feature to the model outputs.

To date, studies investigating the association between body composition and reflux esophagitis (RE) using CT-based third lumbar vertebra cross-sectional imaging remain limited, particularly regarding model interpretability. Therefore, this study aims to develop an accurate classification model for RE using machine learning techniques, explore the relationships between muscle and fat composition along with other clinical factors, and identify the most effective classification model. Finally, Shapley Additive exPlanations (SHAP) values will be utilized for visualization to enhance model interpretability.

## 2 Materials and methods

### 2.1 Study population

This retrospective, cross-sectional study enrolled individuals who underwent routine health examinations at Zhongda Hospital Affiliated with Southeast University from January 2023 to February 2024. The study population consisted of individuals who voluntarily selected advanced health examination packages, which routinely included gastroscopy and non-contrast abdominal CT as part of comprehensive clinical assessments. The study adhered to the ethical principles of the Declaration of Helsinki and was approved by the Ethics Committee of Zhongda Hospital, Southeast University (Approval No.: 2023ZDSYLL350-P01), with informed consent waived for all participants.

The inclusion criteria were: (1) Participants aged 18 years or older; (2) Underwent both abdominal CT and gastroscopy; (3) Gastroscopy findings indicating chronic gastritis, reflux esophagitis or hiatal hernia. Exclusion criteria were as follows: (1) History of malignant tumors; (2) History of gastric or esophageal surgery; (3) Diagnosis of inflammatory bowel disease; (4) Stage 5 chronic kidney disease requiring regular hemodialysis; (5) Paralysis, prolonged bed rest, or other physical disabilities; (6) Metabolic disorders affecting basal metabolism, such as thyroid dysfunction, and (7) Unavailable CT images or missing clinical data.

Based on gastroscopic findings, participants were categorized into two groups. The RE group was defined as individuals with endoscopically confirmed esophageal mucosal injury (reflux esophagitis), with or without the presence of a hiatal hernia. The matched health control (MHC) group, representing the non-reflux esophagitis (non-RE) group, was defined as individuals without reflux esophagitis, although they might have been diagnosed with chronic gastritis or hiatal hernia.

### 2.2 Data collection and preprocessing

This study obtained patient demographic and clinical information from the electronic medical record system, including sex, age, BMI and history of smoking, alcohol consumption, hypertension, diabetes and coronary heart disease. Gastroscopy images were retrieved from the endoscopy database and confirmed by two senior gastroenterologists, identifying conditions such as RE, chronic gastritis, and hiatal hernia. Laboratory test results, including blood glucose, total cholesterol (TC), triglycerides (TG), high-density lipoprotein cholesterol (HDL), low-density lipoprotein cholesterol (LDL), apolipoprotein A1 (ApoA1), apolipoprotein B (ApoB), and lipoprotein (a) [Lp(a)], were extracted from the clinical laboratory system.

Abdominal CT images were retrieved from the imaging management system, and cross-sectional slices at the third lumbar vertebra (L3) level were selected for body composition analysis and fatty liver assessment. All slices were manually selected by two trained researchers, and image analysis was performed using Slice-O-matic software (version 4.3, Tomovision, Montreal, QC, Canada). Muscle and fat tissues were segmented based on their anatomical location and Hounsfield unit (HU) values, distinguishing the following four regions: (1) skeletal muscle (SM, -29 to +150 HU), (2) intermuscular adipose tissue (IMAT, -190 to -30 HU), (3) subcutaneous adipose tissue (SAT, -190 to -30 HU), and (4) visceral adipose tissue (VAT, -150 to -50 HU) (Prado et al., 2008). The average values from two separate segmentations were used for data analysis.

Body composition indices were calculated using the following formulas: (1) Skeletal Muscle Index (SMI, cm²/m²) = Skeletal Muscle Area (SMA)/height²; (2) Fat Mass (FM, kg) = 0.042 × L3 fat area + 11.2; (3) Fat-Free Mass (FFM, kg) = 0.3 × L3 skeletal muscle area + 6.06; (4) Fat-Free Mass Index (FFMI, kg/m²) = FFM/height²; (5) Fat Mass Index (FMI, kg/m²) = FM/height²; and (6) Visceral-to-Subcutaneous Fat Ratio (VSR) = VAT/SAT (Caan et al., 2018; Tonnesen et al., 2024). All collected data were incorporated into the model development. To preserve the distribution of the target variable (RE), the dataset was split into a training set (80% of the dataset) and a validation set (20% of the dataset) using stratified random sampling based on the RE outcome.

### 2.3 Definitions

(1) General Obesity and Visceral Fat Obesity: According to the Asia-Pacific criteria established by the World Health Organization Western Pacific Region, obesity is defined as BMI ≥ 25 kg/m², while non-obesity is defined as BMI < 25 kg/m². Visceral fat obesity (VFO) is determined by the visceral-to-subcutaneous fat ratio (VSR), with VFO defined as VSR ≥ 1.0 in men and VSR ≥ 0.5 in women (Kim et al., 2023).

(2) Body Composition Thresholds: Low skeletal muscle mass index (SMI) is defined as SMI ≤ 38.5 cm²/m² in women and SMI ≤ 52.4 cm²/m² in men (Raoul et al., 2024; Prado et al., 2008). Low fat-free mass index (FFMI) is defined as FFMI ≤ 15 kg/m² in women and FFMI ≤ 17 kg/m² in men. High fat mass index (FMI) is defined as FMI ≥ 13 kg/m² in women and FMI ≥ 9 kg/m² in men (Caan et al., 2018).

(3) Normal Lipid Profile Reference Values: Total cholesterol (TC) < 6.2 mmol/L, triglycerides (TG) < 2.3 mmol/L, high-density lipoprotein cholesterol (HDL) ≥ 1 mmol/L, low-density lipoprotein cholesterol (LDL) < 3.4 mmol/L, apolipoprotein A1 (ApoA1) ≥ 1.2 mmol/L, apolipoprotein B (ApoB) ≤ 1.2 mmol/L, and lipoprotein (a) [Lp(a)] ≤ 300 mg/L.

### 2.4 Model development

This study consisted of two main stages: (1) feature selection and (2) model development. During the feature selection stage, a Sequential Forward Selection (SFS) strategy based on Random Forest feature importance ranking was applied to determine the optimal number of variables. Feature selection was conducted exclusively within the training set, and at each step model performance was evaluated using stratified five-fold cross-validation rather than the hold-out validation set, to ensure robustness and avoid information leakage. Based on the selected features, six machine learning models were developed and compared: Random Forest (RF), Extreme Gradient Boosting (XGBoost), Logistic Regression (LR), K-Nearest Neighbors (KNN), Support Vector Machine (SVM), and Artificial Neural Network (ANN).

### 2.5 Feature selection

Feature importance scores (VIS) were calculated using the feature importances attribute of the RF classifier, based on the Gini index. To reduce random variability, the random seed was fixed and the Random Forest model was trained multiple times, with the Gini importance values averaged across runs. All features were then ranked in descending order according to their VIS. Following the importance ranking, features were sequentially added to the model in a forward manner. Starting from the top-ranked feature, the top k features (k = 1…N) were incrementally included. At each step, model performance was evaluated using stratified five-fold cross-validation within the training set by calculating the area under the ROC curve (AUC), and model error was defined as 1 − AUC. The optimal feature subset was determined by identifying the number of features corresponding to the minimum model error. The final selected feature subset was then applied once to the independent validation set for final performance evaluation.

### 2.6 Machine learning model development

Based on stratified data partitioning, six machine learning algorithms were developed, including RF, XGBoost, LR, KNN, SVM, ANN. To ensure optimal model performance and fair comparison across algorithms, a stratified 5-fold cross-validation based Grid Search framework was implemented for hyperparameter optimization exclusively within the training set. For the core Random Forest model, the optimization process identified an ensemble of 500 decision trees (n_estimators = 500), with the maximum depth of each tree limited to five (max_depth = 5). For models sensitive to feature scaling, including LR, KNN, SVM, and ANN, preprocessing was performed using pipelines incorporating a StandardScaler within the cross-validation procedure to prevent data leakage. In addition, cost-sensitive learning strategies (adjustment of class_weight or scale_pos_weight) were applied where appropriate to mitigate the impact of class imbalance. Through iterative refinement within predefined hyperparameter search spaces, the optimal configuration for each algorithm was ultimately determined.

Final model performance was evaluated only once on an independent hold-out validation set that was not involved in feature selection or model tuning, using the area under the receiver operating characteristic curve (AUROC), along with accuracy, recall, F1 score, and specificity. To analyze the decision-making mechanism of the models, the SHAP framework was applied to interpret the best-performing Random Forest model. SHAP values were computed using Tree Explainer, with summary plots highlighting the positive global contributions of IMAT, VSR, and VAT to reflux esophagitis likelihood. Dependence plots illustrated the nonlinear relationships between variables and classification outcomes, validating the clinical relevance of threshold binarization. SHAP values not only quantified the contribution of individual variables but also demonstrated their overall impact on the model’s discriminative performance.

### 2.7 Statistical analysis

Statistical analyses were performed using Python version 3.12.8 and SPSS version 26.0, incorporating libraries such as scikit-learn, pandas, seaborn, numpy, and shap. Continuous variables following a normal distribution were expressed as mean ± standard deviation ( $\bar{x}$ ± *s*), with intergroup differences analyzed using t-tests and one-way ANOVA. Skewed continuous variables were presented as median (Q1, Q3), and group comparisons were conducted using the Mann-Whitney U test with Bonferroni correction. Categorical variables were reported as counts (percentages) and analyzed using the *χ*² test or Fisher’s exact test. A two-sided *P*-value < 0.05 was considered statistically significant.

## 3 Results

### 3.1 Study population

A total of 324 subjects were included in the study, with 189 in the healthy control group (MHC; non-RE group) (87 males, 102 females; mean age 56.9 ± 13.1 years) and 135 in the reflux esophagitis group (RE) (104 males, 31 females; mean age 59.9 ± 12.6 years).

Regarding clinical characteristics, patients in the RE group had significantly higher age, smoking rates, alcohol consumption, BMI, and obesity prevalence compared to the healthy control group (*P* < 0.05). In laboratory tests, HDL levels were significantly lower in the RE group (*P* = 0.005), while ApoB levels were higher but did not reach statistical significance (*P* = 0.060). There were no significant differences between the two groups in hypertension history, diabetes history, fatty liver history, TG, TC, LDL, ApoA1, and Lp(a) levels (all *P* > 0.05, **Table 1**).

**Table 1:** Baseline data comparison between the RE group and the MHC group.

Characteristics	MHC (*n* = 189)	RE (*n* = 135)	*P* value
Age (years, $\bar{x}$ ± *s*)	56.9 ± 13.1	59.9 ± 12.6	0.035
Male, *n* (%)	87 (46)	104 (77)	<0.001
Smoking, *n* (%)	20 (10.6)	28 (20.7)	0.011
Drinking, *n* (%)	16 (8.5)	28 (20.7)	0.001
Hypertension, *n* (%)	63 (33.3)	57 (42.2)	0.102
Diabetes, *n* (%)	25 (13.2)	15 (11.1)	0.568
BMI, kg/m²	24.08 ± 3.20	25.04 ± 3.09	0.018
Obesity, *n* (%)	68 (36)	71 (52.6)	0.003
Fatty liver, *n* (%)	36 (19)	34 (25.2)	0.186
TG, median (Q1, Q3), mmol/L	1.4 (1.0, 2.1)	1.5 (1.0, 2.2)	0.401
TC, median (Q1, Q3), mmol/L	4.7 (4.0, 5.3)	4.7 (3.8, 5.3)	0.798
HDL, median (Q1, Q3), mmol/L	1.3 (1.1, 1.5)	1.2 (1.0, 1.4)	0.005
LDL, median (Q1, Q3), mmol/L	2.6 ± 0.7	2.6 ± 0.9	0.881
ApoA1, median (Q1, Q3), mmol/L	1.2 (1.1, 1.4)	1.2 (1.0, 1.4)	0.715
ApoB, median (Q1, Q3), mmol/L	0.9 (0.7, 1.1)	0.9 (0.8, 1.1)	0.060
Lp(a), median (Q1, Q3), mg/L	136 (61, 246)	114 (53, 236)	0.264

Data are presented as median (interquartile range) or number (%). MHC, matched healthy controls; BMI, body mass index; TC, total cholesterol; TG, triglycerides; HDL, high-density lipoprotein cholesterol; LDL, low-density lipoprotein cholesterol; ApoA1, apolipoprotein A1; ApoB, apolipoprotein B; Lp(a), lipoprotein(a).

For body composition analysis, the prevalence of low SMI was significantly higher in the RE group compared to the healthy control group (52.6% vs. 36.5%, *P* = 0.004). In fat distribution analysis based on CT imaging, the RE group showed significantly higher IMAT, FM, and VAT compared to the healthy control group (all *P* < 0.05), while no significant differences were observed in SAT, VSR, and VFO (all *P* > 0.05, **Table 2**).

**Table 2:** Comparison of body composition analysis in CT images between the RE group and the MHC group.

Characteristics	MHC (*n* = 189)	RE (*n* = 135)	*P* value
Low SMI, *n* (%)	69 (36.5)	71(52.6)	0.004
SMI, median (Q1, Q3), cm²/m²			
Male	53.0 (47.8, 57.8)	52.0 (48.1, 57.5)	0.750
Female	40.1 (37.1, 43.1)	41.0 (35.4, 45.6)	0.966
IMAT, median (Q1, Q3), cm²	6.9 (4.9, 10.0)	9.4 (6.3, 13.7)	<0.001
FM, median (Q1, Q3), kg)	23.0 (20.2, 26.0)	24.6 (22.0, 27.7)	0.006
SAT, median (Q1, Q3), cm²	133.7 (99.5, 168.1)	131.7 (105.6, 169.6)	0.860
VAT (cm², $\bar{x}$ ± *s*)	132.8 ± 66.4	162.1 ± 69.4	<0.001
VSR			
Male	1.3 (1.0, 1.8)	1.3 (1.0, 1.9)	0.727
Female	0.7 (0.5, 0.9)	0.6 (0.4, 1.0)	0.749
VFO, *n* (%)	136 (72)	106 (78.5)	0.181

RE, Reflux esophagitis; SMI, skeletal muscle index; FM, total fat mass; SAT, subcutaneous fat; VAT, visceral fat; VSR, visceral-to-subcutaneous fat ratio; IMAT, intermuscular fat; VFO, visceral fat obesity.

Each patient had 26 clinical features, and they were randomly assigned to either a training set (*n* = 259) or a validation set (*n* = 65). There were no significant differences in the incidence of RE and clinical characteristics between the training and validation sets (all *P* > 0.05).

### 3.2 Feature selection

**Figure 1:**
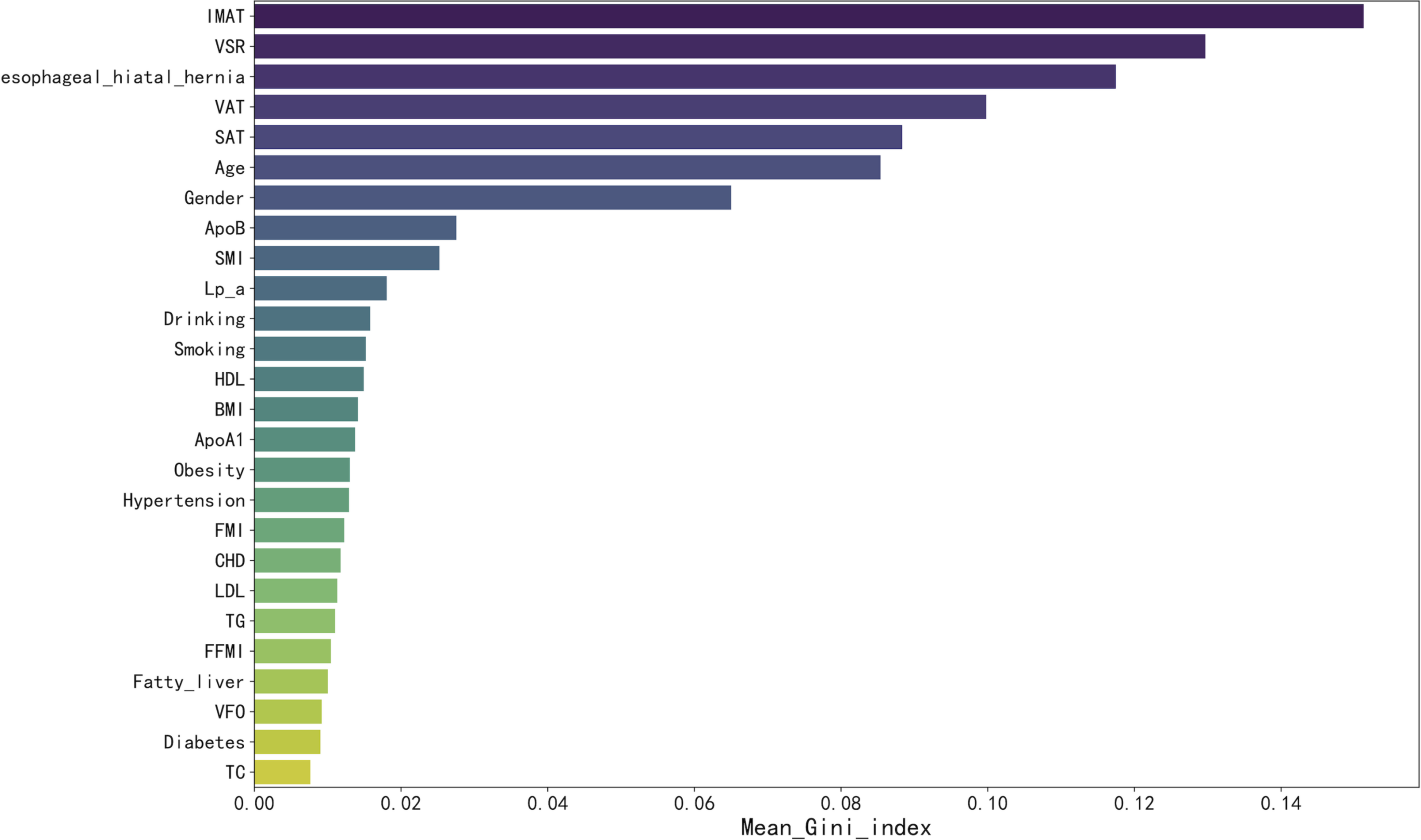
Importance matrix plot of random forest algorithm based on the training set. IMAT, intermuscular fat; VSR, visceral-to-subcutaneous fat ratio; VAT, visceral fat; SAT, subcutaneous fat; ApoB, apolipoprotein B; SMI, skeletal muscle index; Lp(a), lipoprotein(a); HDL, high-density lipoprotein cholesterol; BMI, body mass index; ApoA1, apolipoprotein A1; FMI, Fat Mass Index; CHD, Coronary Heart Disease; LDL, low-density lipoprotein cholesterol; TG, triglycerides; FFMI, Fat-Free Mass Index;VFO, visceral fat obesity; TC, total cholesterol.

**Figure 2:**
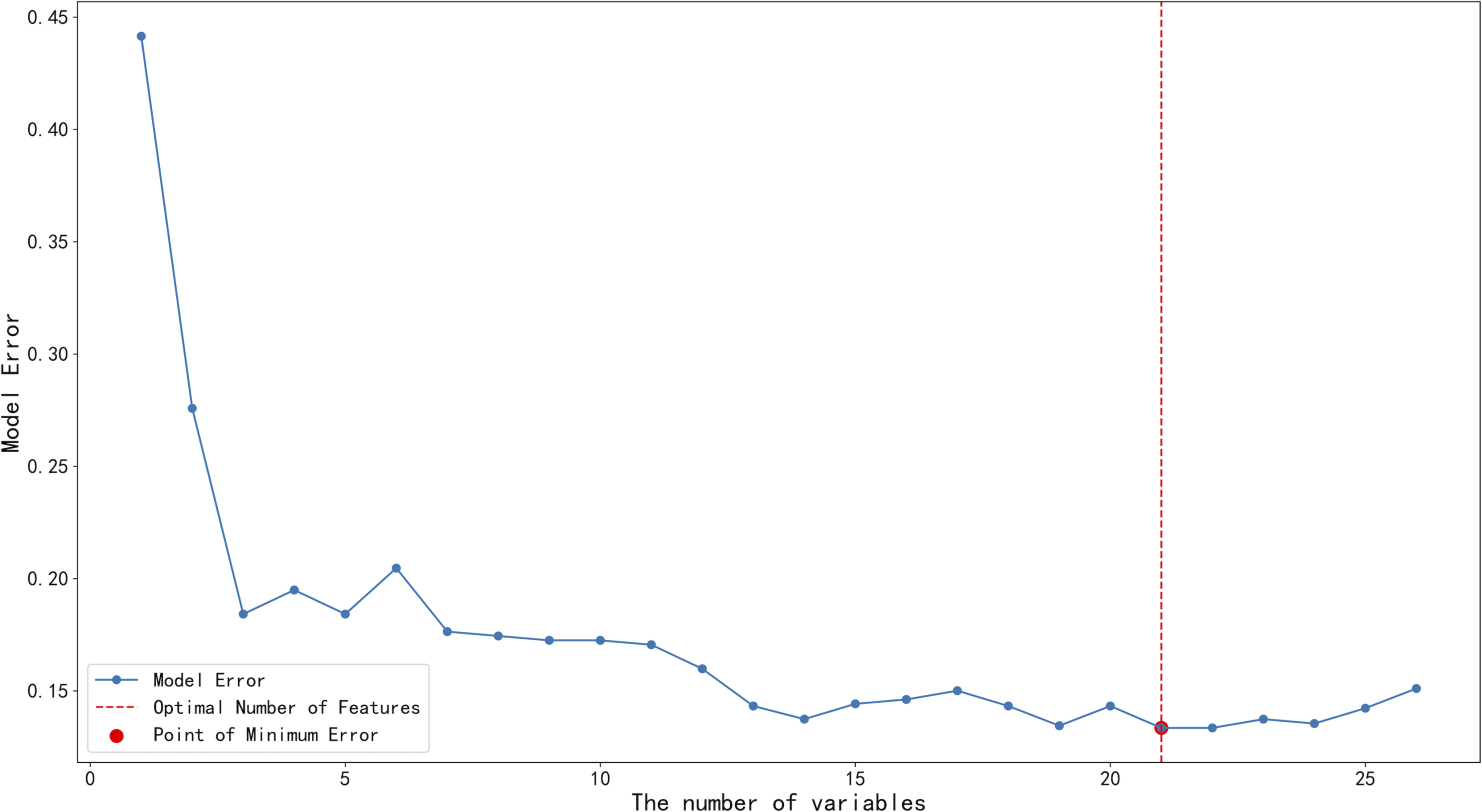
Feature selection using sequential forward selection based on random forest importance ranking. Feature selection using a sequential forward selection strategy based on Random Forest feature importance. Variable importance scores (VIS) were obtained from a Random Forest model and ranked in descending order. Features were then incrementally included in the model according to their VIS ranking. For each feature subset, model performance was evaluated using the area under the ROC curve (AUC), and model error was defined as 1 − AUC. The optimal number of variables (22 variables) was determined by the minimum model error, indicated by the red circle.

Feature importance scores for each variable were computed using the Random Forest (RF) algorithm and ranked in descending order, as illustrated in **Figure 1**. Initially, all 26 features were evaluated, and then they were sequentially incorporated into the Sequential Forward Selection (SFS) procedure according to their VIS ranking. Based on the lowest out-of-bag (OOB) error rate within the training set, as shown in **Figure 2**, SFS identified 22 key features. These included six body composition variables: IMAT, VSR, VAT, SMI, SAT, FFMI; ten clinical baseline characteristics: gender, age, BMI, alcohol consumption, obesity, fatty liver, hypertension, smoking, coronary heart disease (CHD), and diabetes; one endoscopic feature: esophageal hiatal hernia; and five laboratory parameters: ApoB, HDL, ApoA1, Lp(a), and LDL.

### 3.3 Development of ML models for RE classification

**Figure 3:**
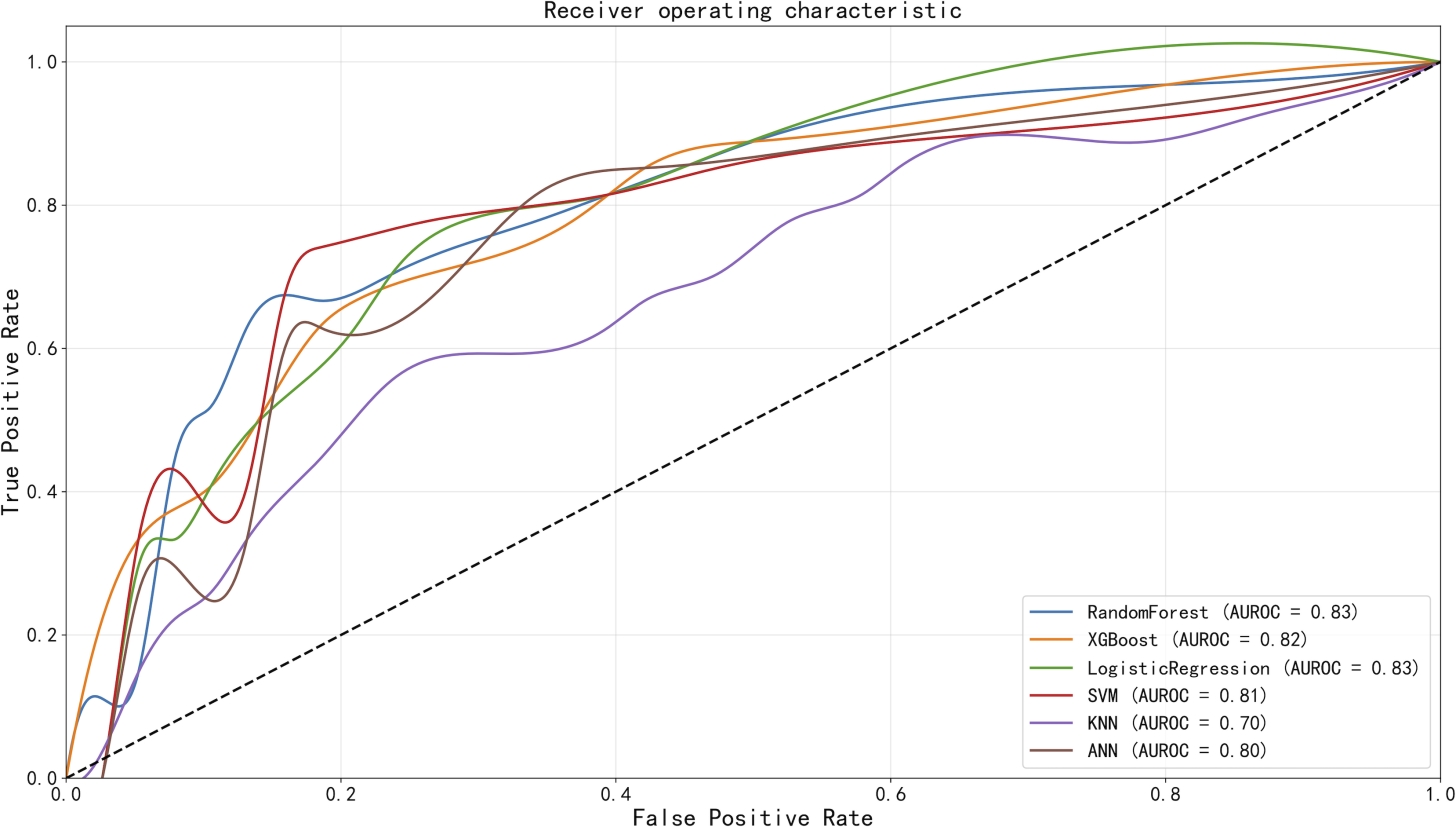
Comparison of AUCs among machine learning models. RF and LR yielded the greatest AUC for RE classification.

After excluding two features with low importance and minimal contribution to the model output, 20 key variables were incorporated into six machine learning models for identifying reflux esophagitis: RF, XGBoost, LR, KNN, SVM, and ANN. **Figure 3** illustrates the performance comparison of these models using ROC curves. Among them, both the Random Forest model (AUC = 0.829, 95% CI: 0.731–0.905) and the Logistic Regression model (AUC = 0.829, 95% CI: 0.736–0.909) demonstrated the best discriminative performance for reflux esophagitis, followed by the XGBoost model (AUC = 0.825, 95% CI: 0.737–0.903), SVM model (AUC = 0.808, 95% CI: 0.716–0.902), ANN model (AUC = 0.798, 95% CI: 0.703–0.888), and KNN model (AUC = 0.701, 95% CI: 0.594–0.799). Detailed performance metrics for all six models are provided in **Table 3**. A comprehensive comparison revealed that the Random Forest model achieved the highest accuracy (0.769) and specificity (0.868), with recall (0.630) and F1 score (0.694) ranking second among the models.

**Table 3:** Model performance metrics.

Models	AUC	Accuracy	Recall	F1 Score	Specificity
Random Forest	0.829	0.769	0.630	0.694	0.868
XGBoost	0.825	0.739	0.630	0.667	0.816
Logistic Regression	0.829	0.723	0.630	0.654	0.790
SVM	0.808	0.769	0.667	0.706	0.842
KNN	0.701	0.677	0.556	0.588	0.763
ANN	0.798	0.754	0.593	0.667	0.868

AUC, area under curve; XGBoost, extreme gradient boosting; SVM, support vector machine; KNN, k-nearest neighbors; ANN, artificial neural network; TN, True negative; TP, True positive; FN, False negative; FP, False positive. Accuracy is computed based on the total number of correct predictions defined as: $\frac{TP+TN}{TP+FN+TN+FP}$; Recall is the ratio of correctly predicted positive observations to all actual positives, defined as: $\frac{TP}{TP+FN}$; Specificity is the ratio of correctly predicted negative observations to all actual negatives, defined as: $\frac{TN}{TN+FP}$.

### 3.4 SHAP value visualization

**Figure 4:**
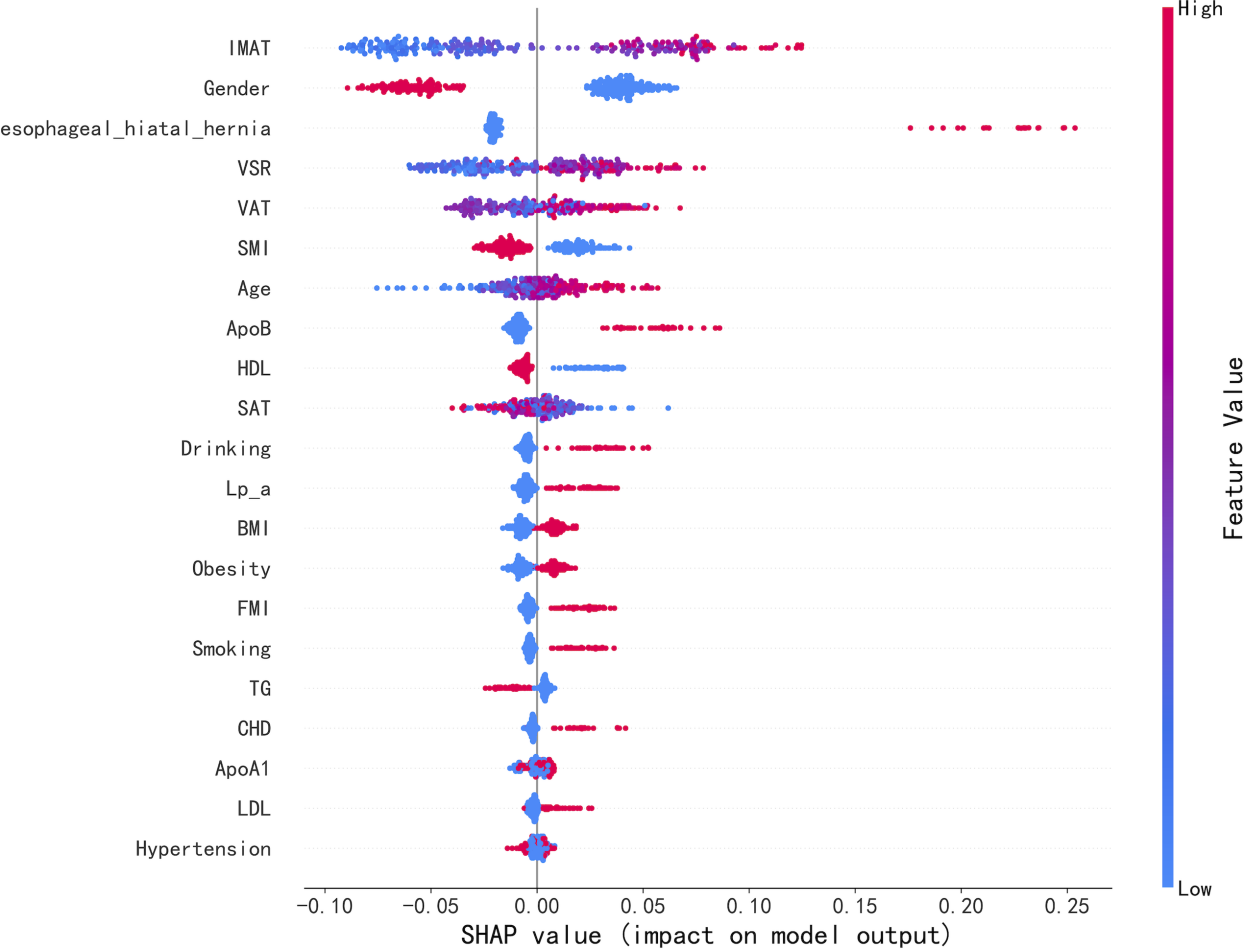
SHAP summary plot of the top 20 features of the RF model. The higher the SHAP value of a feature, the higher the likelihood of the presence of Reflux esophagitis. The parameters are ranked from top to bottom by their contribution to the model output. Feature values are represented by the colour gradient. Higher SHAP values indicate the association of parameters with a higher likelihood of RE presence. IMAT, intermuscular fat; VSR, visceral-to-subcutaneous fat ratio; VAT, visceral fat; SAT, subcutaneous fat; ApoB, apolipoprotein B; SMI, skeletal muscle index; Lp(a), lipoprotein(a); HDL, high-density lipoprotein cholesterol; BMI, body mass index; ApoA1, apolipoprotein A1; FMI, Fat Mass Index; CHD, Coronary Heart Disease; LDL, low-density lipoprotein cholesterol; TG, triglycerides; FFMI, Fat-Free Mass Index;VFO, visceral fat obesity; TC, total cholesterol.

**Figure 5:**
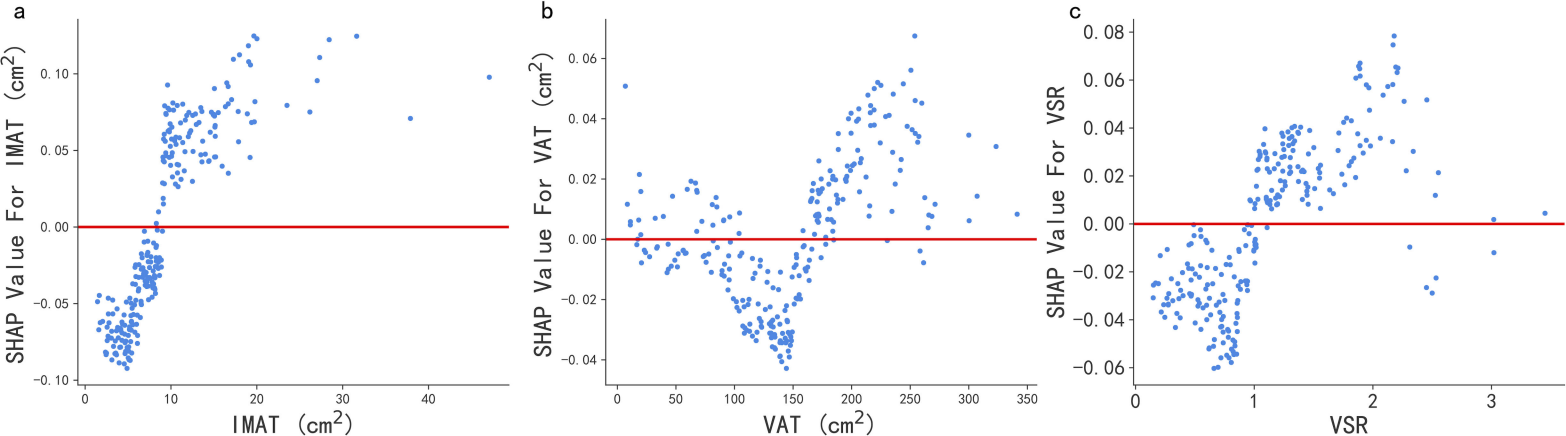
SHAP dependence plot of the RF model. The SHAP dependence plot shows how a single feature affects the output of the RF classification model. SHAP values for specific features exceed zero, representing an increased likelihood of Reflux esophagitis. IMAT, intermuscular fat; VSR, visceral-to-subcutaneous fat ratio; VAT, visceral fat.

To identify the features with the greatest impact on the Random Forest model, we generated a SHAP summary plot (**Figure 4**) for the top 20 features of the classification model. This plot illustrates the relationship between feature values and their corresponding SHAP values within the training set. When the SHAP value for a particular feature exceeds zero at a given value, it indicates an increased likelihood of reflux esophagitis (**Figure 5**).

## 4 Discussion

In this retrospective, cross-sectional study, we developed and validated a series of machine learning models to classify the occurrence of reflux esophagitis. By employing SFS, we identified 22 key factors associated with reflux esophagitis from 26 variables, including body composition and other clinical features, which were then incorporated into the model. Both the Random Forest and Logistic Regression models achieved the highest AUC of 0.829, with the Random Forest model demonstrating excellent discriminative ability and unique interpretative advantages among all the models tested. In the Random Forest importance matrix and SHAP summary plots, four out of the top six features were body composition variables, indicating that body composition plays a crucial role in identifying reflux esophagitis. This study substantiates the value of body composition data, a value that had not been previously demonstrated in the clinical identification of reflux esophagitis.

In this study, in addition to factors previously identified as associated with reflux esophagitis (including age, gender, esophageal hiatal hernia, VAT, BMI, smoking, and alcohol consumption), the remaining variables represent novel indicators for the presence of RE, mainly including IMAT, VSR, SMI, SAT, and lipid indices. In established Random Forest model, both the feature importance matrix and the SHAP summary plot demonstrated that IMAT is the most important feature, indicating that IMAT is the most valuable factor in identifying reflux esophagitis. Furthermore, the SHAP dependence plot for individual features shows that higher IMAT values are associated with an increased probability of the presence of RE. In one of our previous studies, it was also confirmed that IMAT levels are significantly higher in the RE population than in the healthy control group. IMAT (intermuscular adipose tissue) is an ectopic fat accumulation located within the muscle interstitium (i.e., outside muscle fibers) and originates from fat precursor cells (FAPs); it is associated with the loss of skeletal muscle strength and physical function (Takahashi et al., 2023). The underlying mechanism may be that IMAT leads to a decrease in esophageal muscle strength, making reflux more likely, which is associated with reflux esophagitis. Therefore, we speculate that controlling the increase in IMAT may help mitigate the likelihood of RE. Recently, a mouse study suggested that GIP receptor antagonists can inhibit the formation of IMAT and enhance muscle activity (Takahashi et al., 2023), leading us to hypothesize that GIP receptor antagonists might indirectly reduce reflux and alleviate the symptoms of reflux esophagitis. Two previous cross-sectional studies have demonstrated that protein and alcohol intake, as well as serum cholesterol levels, are positively associated with IMAT content (Kazibwe et al., 2023; Sjoholm et al., 2016). A meta-regression analysis further revealed that exercise interventions—particularly moderate-intensity aerobic exercise, either alone or in combination with resistance training, can reduce IMAT levels to a certain extent (Tunon-Suarez et al., 2021). Therefore, in clinical practice, IMAT levels may be modulated through dietary modification and exercise management, both of which are feasible and practical approaches. Notably, a recent review has reported that aging is associated with increased adipogenesis of FAPs and accumulation of IMAT (Flores-Opazo et al., 2024). In our established model, age emerged as the most important discriminative factor after body composition parameters, which is consistent with previous findings. This observation may partly explain the higher susceptibility to RE in older individuals and further supports our conclusion that IMAT is the most important indicator of reflux esophagitis. Although the pathophysiological mechanisms underlying IMAT accumulation and its role in the pathophysiology of reflux esophagitis remain to be fully elucidated, the existing evidence provides a practical basis for clinical management and intervention research from a body composition perspective.

In our established Random Forest model, among the body composition features, VAT and VSR also emerged as significant indicators of reflux esophagitis, highlighting the negative impact of visceral fat obesity on RE. An early cross-sectional study indicated that metabolic syndrome (MS), characterized by visceral obesity, was associated with RE (OR = 1.42, 95% CI: 1.26–1.60), and further analysis of MS components revealed that VAT measured by abdominal CT was an independent associated factor for RE (OR = 1.61, 95% CI: 1.10–2.36) (Chung et al., 2008). A recent large cohort study also demonstrated that an increase in VAT is associated with a higher likelihood (Han et al., 2024). These findings are consistent with our study’s conclusions. Both VAT and VSR represent high levels of visceral fat, and their role as associated factors for reflux esophagitis is likely due to excessive visceral fat, abdominal obesity and elevated intra-abdominal pressure (Collings et al., 2003), which collectively promote gastroesophageal reflux.

This study demonstrated that SMI holds significant discriminative importance for RE in the random forest model. In a cross-sectional study conducted in Korea, limb skeletal muscle mass measured by bioelectrical impedance analysis (BIA) and the skeletal muscle index used to define sarcopenia confirmed an association between sarcopenia and gastroesophageal reflux disease (GERD) (Kim et al., 2019). Additionally, a bidirectional two-sample Mendelian randomization analysis verified a positive correlation between low skeletal muscle mass and GERD (Hu et al., 2024). These findings are consistent with our results, which indicate that a low SMI is a significant associated factor for RE. The primary mechanism may involve insufficient muscle strength in the distal esophagus, leading to reduced lower esophageal sphincter (LES) pressure and subsequent reflux of gastric contents into the esophagus (Hu et al., 2024). Furthermore, inadequate paraspinal muscle strength to maintain normal spinal alignment may result in increased intra-abdominal pressure, thereby contributing to the pathogenesis of GERD (Imagama et al., 2012).

It is noteworthy that while previous studies have primarily focused on the correlation between muscle mass and gastroesophageal reflux, our feature importance matrix and SHAP summary plots indicate that SMI is not the most influential body composition parameter; instead, IMAT ranks highest, followed by VAT. We believe there are two reasons for this observation. First, obesity is closely related to low muscle mass and is often accompanied by high levels of VAT and IMAT, with the increase in IMAT typically preceding the decline in SMI (Li et al., 2022). Thus, in the initial clinical stages of reflux esophagitis, an increase in IMAT may be the predominant change without an accompanying reduction in SMI. Second, studies linking low SMI to gastroesophageal reflux have mainly involved elderly populations, where reduced SMI leads to insufficient back muscle strength (Imagama et al., 2012). Therefore, the negative impact of low SMI on RE may be more pronounced in older individuals. In clinical practice, as the incidence of RE among middle-aged and younger individuals continues to rise, IMAT and VAT could serve as critical potential markers for the early identification of RE.

Interestingly, the linear model (Logistic Regression, LR) and the nonlinear ensemble model (Random Forest, RF) demonstrated comparable discriminative performance in this study (AUC = 0.83). This performance equivalence reflects an algorithmic consensus, suggesting that the associations between the major features and the presence of RE are characterized by robust and largely monotonic effects, which both linear and non-linear algorithms can effectively capture. This mutual validation strengthens the reliability of our findings. In the final stage of the analysis, the RF model was further interpreted using SHAP visualization. One of the main advantages of SHAP summary plots is their ability to rank features according to their overall contribution to model outputs, enabling rapid assessment of the relative importance of individual variables and identification of key factors associated with RE. Compared with the fixed coefficients of LR, SHAP provides a more intuitive and mathematically rigorous framework for global feature attribution. Moreover, SHAP dependence analyses revealed that the RF model was able to capture nonlinear threshold effects and complex interactions among variables. While LR efficiently captures global linear trends, its core assumption is that the association increases at a constant slope. In contrast, RF automatically identifies synergistic effects without manual feature engineering. As demonstrated in our SHAP dependence plots (**Figure 5**), identical values of VAT exhibit significant “vertical dispersion,” meaning that the impact of VAT on the presence of RE depends on the context of other variables, such as gender or IMAT. Compared with the fixed odds-ratio assumption of LR, RF can automatically identify such synergistic association patterns. Consequently, SHAP derived explanations may facilitate individualized assessment and personalized clinical decision making, providing a more granular understanding than traditional statistical models.

In conclusion, this study highlights the potential of machine learning algorithms in developing classification models for RE and offers several advantages over previous studies. First, this is the first study to incorporate multiple factors, including fat and muscle composition, into an RE discriminative model. It utilizes computed tomography (CT) to analyze muscle and fat content at the L3 level, a method previously established as the gold standard for body composition assessment alongside magnetic resonance imaging (MRI) (Li et al., 2022). Second, this study specifically focuses on RE, avoiding the subjectivity associated with questionnaire-based studies that define GERD as the research target. Finally, many prior studies lacked rigorous model validation and performance assessment, which limited their ability to confirm classification accuracy. In contrast, this study employs a comprehensive machine learning framework with robust internal validation. Our results confirm that machine learning models offer distinct advantages in managing high-dimensional clinical data and capturing intricate non-linear relationships compared to traditional logistic regression, a conclusion consistent with existing literature (Jiang et al., 2017; Rajkomar et al., 2019).

This study has several limitations. First, it is based on single-center data with a relatively small sample size. This limitation primarily stems from our manual segmentation of abdominal CT images to quantify muscle and fat composition, rather than utilizing deep learning algorithms for automatic segmentation and three-dimensional quantification. The labor-intensive nature of manual segmentation restricted our ability to include a larger sample. We are actively developing an automatic abdominal CT segmentation algorithm to address this issue. Second, patient characteristics were manually entered into the system. To facilitate the application of machine learning models, we are developing an online networked tool integrated with the electronic medical record (EMR) system. This tool will automatically import relevant clinical parameters from EMRs alongside manually inputted body composition data, enabling real-time assessment of reflux esophagitis (RE) likelihood. Third, model performance evaluation in the present study relied on a hold-out validation strategy. Although model parameters were optimized using 5-fold cross-validation, the hold-out split may still introduce variability into the final performance estimates. Future studies should consider adopting repeated or nested cross-validation approaches to more comprehensively quantify the impact of data partitioning on model stability. In addition, beyond internal validation, the lack of external validation may limit the generalizability of the findings, and further multicenter studies are warranted to confirm the robustness of the model. Finally, as this study was based on a cross-sectional design, all CT examinations and endoscopic assessments were performed during the same health examination, and no longitudinal follow-up data were available. Therefore, the temporal sequence between variables could not be established, and causal relationships between the associated variables and RE could not be further determined. Future prospective studies should systematically collect body composition parameters at baseline and record incident RE through long-term follow-up, thereby clarifying the temporal relationship between exposures and outcomes. On this basis, standardized gastroesophageal reflux disease questionnaires, such as GERD-Q, should be incorporated, and the study population should be expanded to include patients with non-erosive reflux disease as well as reflux esophagitis. In addition, clinical features such as muscle function and muscle strength should be integrated to further evaluate the independent predictive value of relevant indicators, thereby enabling the development of more rigorous and clinically applicable risk assessment models. These efforts will help facilitate the transition of machine learning–based approaches from disease identification toward risk stratification and prediction, and provide support for individualized management strategies.

Future work will integrate our machine learning model with electronic health record (EHR) systems to adjust associated factors for reflux esophagitis in real time. We also aim to develop more advanced machine learning models to enhance model robustness and clinical utility, ultimately supporting personalized management strategies and improved patient outcomes.

## 5 Conclusion

In this study, we identified 22 key factors associated with RE and developed six machine learning models, revealing that body composition plays a crucial role in identifying the presence of reflux esophagitis. This finding supports the value of body composition analysis for enhanced clinical screening and the development of personalized management strategies for patients.

## Data Availability

The raw data supporting the conclusions of this article will be made available by the authors, without undue reservation.
